# Frederick K. Korley receives the 2026 ASCI/Louis W. Sullivan, MD, Award

**DOI:** 10.1172/JCI209122

**Published:** 2026-06-01

The American Society for Clinical Investigation (ASCI) honors Frederick Kofi Korley, MD, PhD (Figure 1), with the 2026 ASCI/Louis W. Sullivan, MD, Award. Dr. Korley is recognized for his pioneering research in acute care diagnostics and therapeutics, and his impactful commitment to training physician-scientists. He is Professor and Associate Chair for Research in Emergency Medicine at the University of Michigan as well as Scientific Director of the Massey TBI [traumatic brain injury] Grand Challenge, sponsored by the University of Michigan Weil Institute. Dr. Korley chairs the American College of Emergency Physicians TBI expert panel and was the 2021 recipient of the Society of Academic Emergency Medicine Mid-Career Investigator Award. He was elected to the ASCI in 2024. ASCI President-Elect Dr. Stephen Y. Chan, Vitalant Chair in Vascular Medicine and Professor of Medicine and Director of the Vascular Medicine Institute at the University of Pittsburgh School of Medicine and UPMC, interviewed Dr. Korley at the AAP/ASCI/APSA Joint Meeting in Chicago in April 2026.

Stephen Y. Chan: Congratulations, Dr. Korley. Can we start off by having you tell us a little bit about yourself, your training program and path, as well as how you came to study the problems that you’re interested in your lab?

Frederick K. Korley: I’m an emergency physician, and what I’m passionate about is taking care of people and finding new ways of taking better care of people. My journey to a research career has been nontraditional. After residency training, I took a job at [Johns] Hopkins as the Director of Simulation Education, and I knew nothing about research. I did that for three years, did a good job, won a teacher of the year award, and enjoyed teaching. But then I realized that in emergency medicine, because [it’s] such a young specialty, the biggest need was the evidence base that backs what we do. I decided that what I really wanted to do was contribute to that evidence base and not just do things because my attending says so but do it because I know the data says so. So I decided to go back to school to get a PhD. I had a KL2 award from the Hopkins Clinical and Translational Science Award (CTSA) [program] and went to the Bloomberg School of Public Health to do a PhD degree. At that time, the thing that was really bothering me in emergency care was how long it took for us to answer the question “Doc, I have chest pain. Am I having a heart attack?” It used to take almost twenty-four hours back then. My PhD thesis focused on high-sensitivity troponin. Actually, it was one of the first studies of high-sensitivity troponin in the country, where we were looking at [whether] high-sensitivity troponins [could] help us improve how quickly we answer that question.

While doing that, I was working in the lab of a proteomics guru, Jennifer Van Eyk, who is currently at Cedars-Sinai and who was an amazing mentor. During one of the lab meetings, I realized that they had discovered some new proteins that were markers of brain injury in sickle cell patients with silent brain ischemia. I got to thinking that these markers are probably just markers of neurodegeneration and could be released from any cause. In emergency medicine, there is just no way to accurately diagnose acute brain injuries. It’s just one of those organs where we have no blood test. Every [other] organ has blood tests; there’s no blood test for brain injury. So we started thinking, “Could these proteins be useful in traumatic brain injury?” That was a pivot ultimately and led to a career of trying to figure out ways to diagnose traumatic brain injury better and to develop new therapies. And that’s what I’ve been focused on for the last several years.

SYC: Wonderful. What factors do you think were most critical to your success thus far?

FKK: Several factors. One was the training: I kid you not when I say that when I walked into my first PhD class, I knew nothing about research. There was just no way I could do it. Taking that time out to get the tools to be trained was critically valuable for me. Mentorship was the other critical thing. I have been incredibly fortunate to have worked with some of the most amazing, brilliant, generous people who have taught someone who knew nothing. Because the classes teach you some stuff, but ultimately a lot of what we learn is based on hands-on practice. [Participating] in environments that nurture training and provide opportunities to do good work and to collaborate with amazing people has also contributed. The last thing I’ll mention is that I am naturally just creative. I am always thinking about, “How can we do it better? Yes, I know this is how we do it today, but how could tomorrow look way better than today?” Those are some of the things I would say have helped me so far.

SYC: That’s really inspiring. What kind of obstacles can you remember that were also in your path towards this success?

FKK: Frankly, one of the biggest obstacles has been me. I don’t know that I’ve faced a ton of external obstacles. And yes, there have been times when some additional funding could have been better, but I think that some of the limitations are [due to] time. There are so many things I want to do, but so little time. The second one is also related, which is the fact that by being an emergency physician with varied interests when it comes to medicine and diseases, I tend to gravitate toward the problems that matter the most. A lot of times it’s not just one, and it’s not just one direction. Whether it’s been an obstacle or an advantage, [I’ve] yet to fully decide, but I think that that has also led me to do [many] different things and not necessarily homing on one single thing, which I think a lot of scientists do. But I’ve enjoyed it all the same.

SYC: What are your future directions in your research and your career?

FKK: We’re very proud of some things we’ve done, which include helping with the discovery and validation of some blood-based biomarkers, contributing to the work that led to FDA approval of the first blood tests that are being used clinically now, working with engineers to create a point-of-care device so we can measure these biomarkers and do it on the cheap. Because a lot of these discoveries can’t go to the people who really need them in low-resource areas if we don’t make them cheap. [We’ve] done work in implementing these biomarkers clinically, and at the University of Michigan for the last year or so, we’ve been using these tests to treat patients clinically, so seeing that full cycle has been awesome. What’s next is doubling down on finding therapies for TBI patients. I’ve been fortunate to be able to lead some clinical trials where we’ve collected a ton of samples and very well phenotyped these patients. And now we’re at the point where we want to do very deep proteome profiling so we can identify druggable targets and ultimately be able to take them into clinical trials so we can make people better.

SYC: What an exciting future. Do you have any lessons learned about your training path and any sort of advice that you would have for trainees?

FKK: In terms of lessons learned, number one is never be afraid of making a mistake. At the beginning of my career, because I grew up in a low-income household and was determined to be successful, I thought failure was always going to be a big distraction. I was really intimidated by failure. But then when I got to the point where I understood that failure is important because you at least have learned that that is not the right path to follow, that became very freeing and allowed me to imagine more, to take bold risks. And that has been very beneficial. So one major thing is not to be paralyzed by fear. I would also say it’s important to stay humble and understand the limitations in your knowledge. I think it’s something that I have done very well, and I’ve understood my limits and built great teams around me, because the best science is done in a multidisciplinary way. The last thing I’ll mention is that it’s good to dream. As a scientist, this is one of the fields where being a dreamer is really important, because the most successful are the most powerful dreamers, because if you dream it enough and if you work hard enough, your dreams can come to life.

SYC: Dr. Korley, thank you very much for spending time with us, and congratulations on this significant Milestone Award.

FKK: Thank you so much.

*The interview has been edited for length and clarity*.

## Figures and Tables

**Figure 1:**
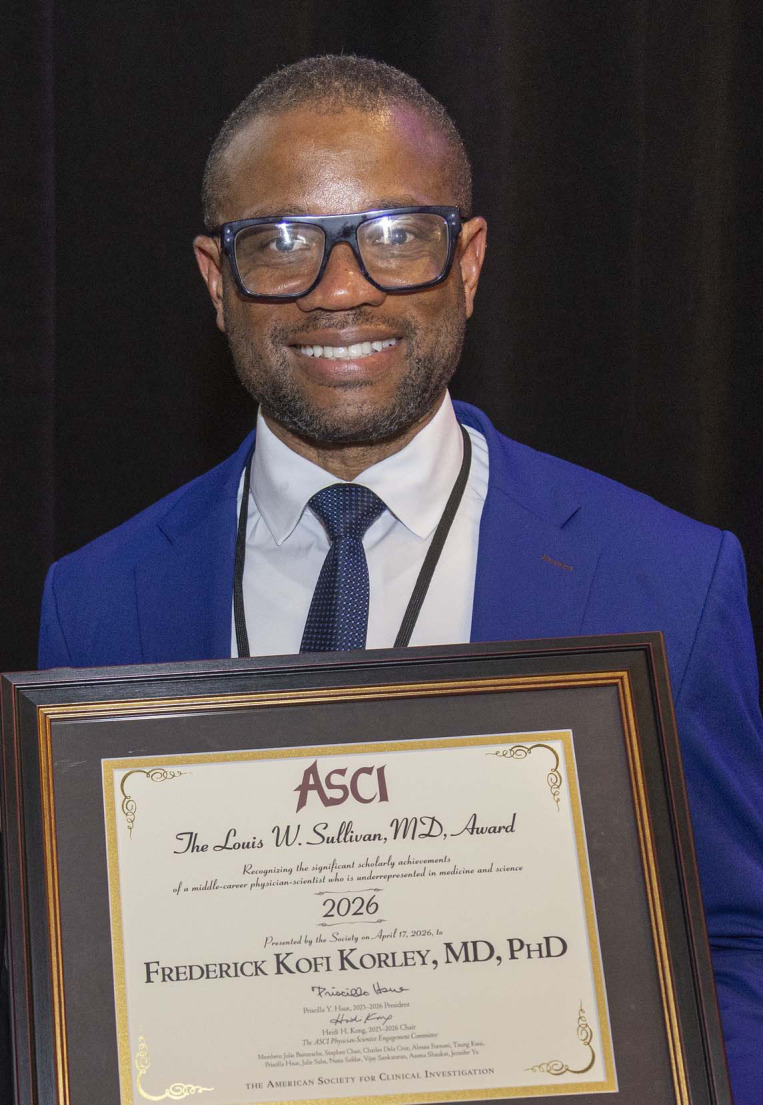
Frederick K. Korley is the recipient of the 2026 Louis W. Sullivan, MD, Award.

## Version 1 06/01/2026

Electronic publication

